# Importance of the traditional food systems for First Nations adults living on reserves in Canada

**DOI:** 10.17269/s41997-020-00353-y

**Published:** 2021-06-28

**Authors:** Malek Batal, Hing Man Chan, Karen Fediuk, Amy Ing, Peter Berti, Tonio Sadik, Louise Johnson-Down

**Affiliations:** 1 Département de nutrition, Faculté de Médecine, Université de Montréal, Pavillon Liliane de Stewart, CP 6128 succ. Centre-Ville, Montréal, QC H3T 1A8 Canada; 2 Centre de recherche en santé publique de l’Université de Montréal et du CIUSS du Centre-sud-de-l’Île-de-Montréal (CReSP), 7101 Avenue du Parc, Montréal, QC H3N 1X7 Canada; 3 Department of Biology, University of Ottawa, 30 Marie Curie, Ottawa, ON K1N 6N5 Canada; 4 First Nations Food, Nutrition and Environment Study, University of Ottawa, 30 Marie Curie, Ottawa, ON K1N 6N5 Canada; 5 HealthBridge Foundation of Canada, 1 Nicholas Street, Suite 1004, Ottawa, ON K1N 7B7 Canada; 6 Assembly of First Nations, 55 Metcalfe Street, Suite 1600, Ottawa, ON K1P 6L5 Canada

**Keywords:** Indigenous, First Nations, Traditional food, Ecozones, Climate change, Autochtones, Premières Nations, Aliments traditionnels, Écozones, Changement climatique

## Abstract

**Objective:**

To describe the traditional food (TF) systems of First Nations in Canada, including intake, barriers and promoters.

**Methods:**

The First Nations Food, Nutrition and Environment Study is a cross-Canada participatory study of First Nations adults below the 60^th^ parallel that obtained data for communities excluded from other national studies. A food frequency questionnaire was used to establish frequency of TF intake (number of days in a year) to allow comparisons across ecozones/regions in Canada. Grams of TF intake were also calculated using frequency multiplied by average portions from 24-h recalls. Closed- and open-ended questions attempted to identify some of the key barriers and concerns regarding TF access and use. Multivariable analyses were run to determine what factors are associated with increased TF consumption.

**Results:**

Across communities, there is a strong preference by adults to have TF in the diet more often. Consumption of land animals was most frequently reported in most ecozones except for the Pacific Maritime and Mixedwood Plains, where fish and plants, respectively, were more frequently consumed. First Nations identified structural and environmental challenges such as development, government regulations and climate change, along with household barriers such as insufficient capital for equipment and transportation, lack of time and absence of a hunter in the household. Multivariable analyses revealed that the highest intake of TF occurred in the Taiga Plains ecozone, and for older individuals and men.

**Conclusion:**

Identifying solutions that empower First Nations at all levels is required to overcome the multiple challenges to the inclusion of TF in the diet.

## Résumé

**Objectifs:**

Décrire les systèmes alimentaires traditionnels (AT) des adultes provenant des Premières Nations du Canada, y compris la consommation, les barrières et les promoteurs.

**Méthode:**

L’Étude sur l’alimentation, la nutrition et l’environnement chez les Premières Nations est une étude participative pancanadienne avec les Premières Nations demeurant dans les réserves au sud du 60^e^ parallèle et qui a permis d’obtenir des données sur les communautés exclues d’autres études nationales. Un questionnaire sur la fréquence des aliments fut utilisé pour établir la fréquence de l’apport en AT (nombre de jours par année) afin de permettre des comparaisons entre les ecozones et régions du Canada. Les grammes d’apport en AT furent également calculés en utilisant la fréquence multipliée par les portions moyennes provenant des rappels de 24 heures. Des questions fermées et ouvertes ont tenté d’identifier certains des principaux obstacles et préoccupations concernant l’accès et l’utilisation des AT. Des analyses multivariées furent effectuées pour identifier quels facteurs sont associés à une consommation plus élevée des AT.

**Résultats:**

Dans toutes les communautés, les adultes préfèrent avoir un apport plus fréquent des AT. La consommation des animaux terrestres a été mentionnée le plus souvent dans la plupart des écozones, sauf dans les Maritimes Pacifiques et les Plaines Boismixtes, où les poissons et les plantes, respectivement, étaient plus fréquemment consommés. Les Premières Nations ont identifié des défis structurels et environnementaux tels que le développement, la réglementation gouvernementale et les changements climatiques, ainsi que des obstacles pour les ménages comme l’insuffisance de capitaux pour l’équipement et le transport, le manque de temps et l’absence de chasseur dans le ménage. Une analyse multivariée a révélé que la consommation la plus élevée d’AT avait lieu dans l’écozone de la Taïga des Plaines, ainsi que pour les personnes plus âgées et les hommes.

**Conclusion:**

Il est important d’identifier des solutions qui renforcent le pouvoir des Premières Nations à surmonter les défis multiples qui entravent la consommation des AT.

## Introduction

Traditional food (TF) has key nutritional, cultural, spiritual and economic values for First Nation Peoples (Willows 2005; Power 2008). For thousands of years, First Nations have relied on food harvesting strategies including hunting, fishing, gathering (e.g., plants, berry picking, maple sap, and root digging) and intensive food production practices such as clam gardens, berry patches and species domestication to procure their TF (Deur and N.J. 2005; Waldram et al. 2006; Murphy et al. 2009). Colonial assimilation policies have led to a dietary transition, resulting in a decline in the availability, quality, safety and access to TF (Egeland and Harrison 2013; Turner et al. 2013). Previous studies have reported that TF use by Indigenous Peoples in Canada is influenced by a multitude of factors, including: environmental factors (ecosystem quality and natural resource management), government regulations that limit hunting and fishing or prohibit sales of TF, development, community factors (location, land access and community programs), interpersonal factors (extended family, social network, sharing, intergenerational influence and learning) and individual factors (preferences, cost, time, skills and convenience) (Chan et al. 2006; Laberge Gaudin et al. 2015; Turner et al. 2013; Power 2008; Willows 2005; Leibovitch Randazzo and Robidoux 2019).

The decreased consumption of TF corresponds with increasing prevalence of obesity and nutrition-related chronic diseases (NRCD) such as type 2 diabetes and cardiovascular disease in First Nations (Ayach and Korda 2010; Batal and Decelles 2019; Haman et al. 2010). Rates of obesity are alarming in First Nations and they have been increasing over time at a faster rate than in the general population of Canada, (Batal and Decelles 2019). This is worrisome as First Nations suffer from higher prevalence of NRCD as compared with Inuit and the general population of Canada, and our understanding of reasons for the differences between First Nations and non-Indigenous Canadians is limited since First Nations living on reserves have been excluded from large national studies such as the Canadian Community Health Survey (CCHS); the First Nations Regional Health Survey is the only other cross-Canada study of First Nations but included more northern communities and did not include an extensive nutrition component (Ayach and Korda 2010; Haman et al. 2010; Batal and Decelles 2019; First Nations Information Governance Centre 2018).

Not only is TF known to improve the intake of many nutrients such as protein, vitamin D, iron and magnesium in Indigenous Peoples, it is often replaced by store-bought market foods (MF) of lesser nutritional content such as low-quality ultra-processed foods (Batal et al. 2018; Batal et al. 2021; Johnson-Down and Egeland 2013). Furthermore, the physical activity involved in the harvesting of TF is an added health benefit and it also contributes to the cultural and personal well-being of First Nations (Willows 2005).

There is a paucity of nationally representative data on diet from First Nations adults in Canada and we need to have a better understanding of the diet, particularly the variety and amount of TF harvested locally, of First Nations living on-reserve in order to develop better policies to help improve the intake of these important foods (Chan et al. 2021). The use of different methods of collecting TF data, such as 24-h recalls and food frequency questionnaires (FFQ), impedes comparability between studies: some report TF intake by days of intake, others by percent of individuals reporting TF consumption, as a source of nutrients, or within a dietary pattern (Batal et al. 2005; Batal et al. 2018; deGonzague et al. 1999; Downs et al. 2009; Doyle et al. 2012; Gendron et al. 2016; Delormier and Kuhnlein 1999; Kuhnlein et al. 2013; Kuhnlein et al. 2001). Twenty-four-hour recalls provide a good estimate of the nutrient and food intake of a group, but single 24-h recalls give only a snapshot of intake in time and thus are not the best method to capture the seasonality of TF harvesting (Willett 2012). FFQ can give better estimates of TF because they capture data over time (Dao et al. 2019). There are limited studies that use FFQ in First Nations populations: Batal et al. (Batal et al. 2005) reported amounts of TF ranging from 183 to 439 g/day using FFQ and provided details on the amounts and variety of these important foods in the Denendeh and Yukon. Delormier and Kuhnlein (1999) describe how often TF were consumed by Cree women (Delormier and Kuhnlein 1999).

Because our objective was to report on individuals’ year-round usual intakes compared across Canada, results from the FFQ will be reported here using 24-h recalls for portion calculation only. Actual TF intakes of foods and nutrients from 24-h recalls are reported elsewhere (Batal et al. 2021). We also explored the barriers and promoters impacting TF intake.

## Methods

**Fig. 1:**
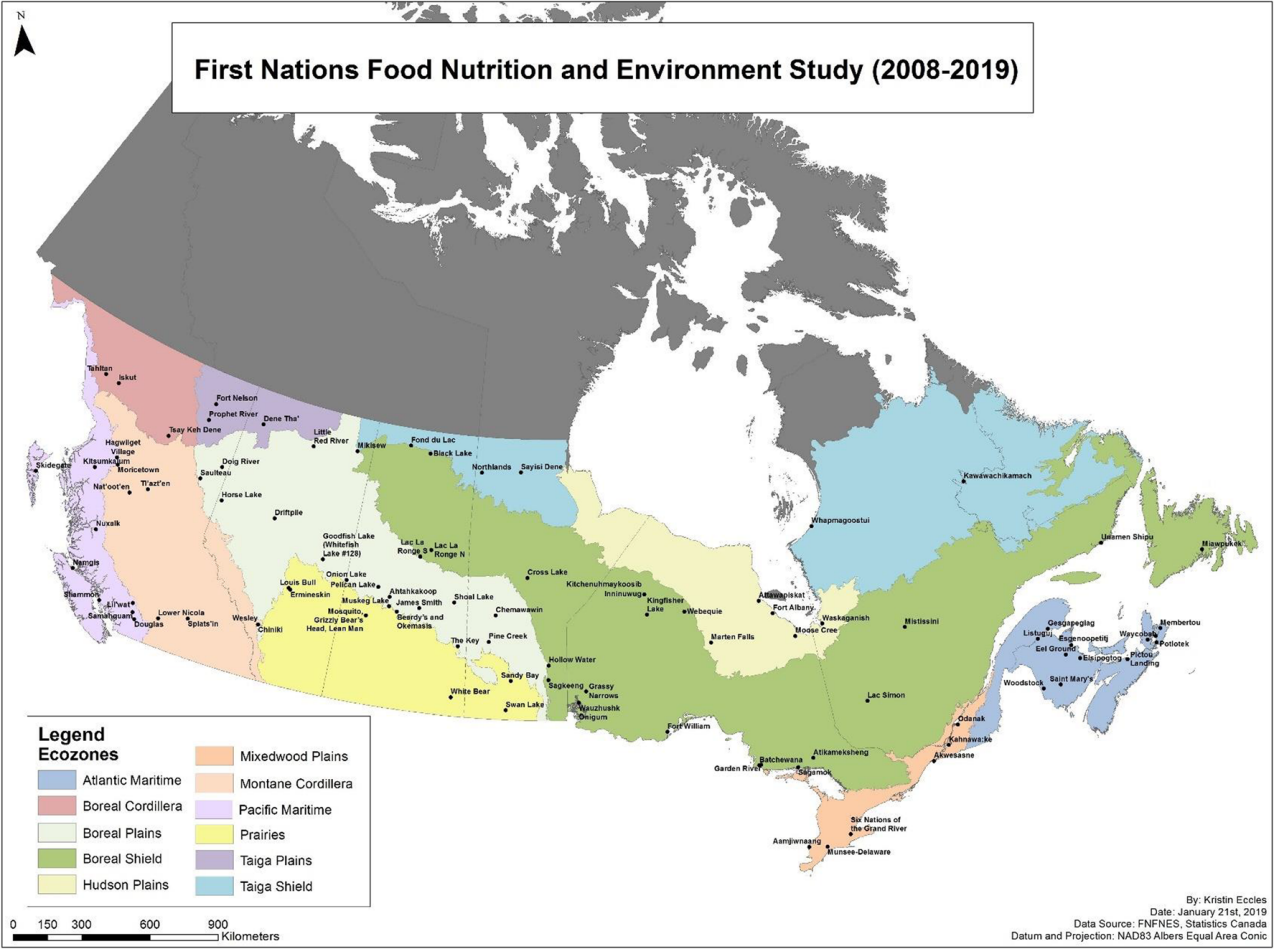
Map of Canada with regions, ecozones and communities participating in the study

The First Nations Food, Nutrition and Environment Study (FNFNES) is a cross-Canada participatory study of First Nations adults living south of the 60^th^ parallel that was undertaken because there were no nationally representative data for this particular population (Chan et al. 2021). Briefly, communities were sampled in each Assembly of First Nations region by Statistics Canada so the sample would be representative of all First Nations adults in the region; second-level randomization took place in selecting households based on household lists provided by the community leadership; and third-level randomization took place within the household to select the adult respondent (Chan et al. 2021). Eleven ecozones (Fig. 1) were identified based on ecosystems (Wiken 1986) whereas regions were mostly provinces, except for Labrador which was included with Quebec, and the Atlantic provinces that were grouped. First Nations principles of Ownership, Control, Access and Possession (OCAP®) were followed (Chan et al. 2021; Schnarch 2004). Informed consent was obtained from all participants (Chan et al. 2021).

Interviews to investigate TF intake and access (i.e., difficult to access meant that they had to go farther to get TF since animals had moved out of the territory due to flooding or other natural disasters) as well as 24-h recalls were conducted by trained community workers under the guidance of trained dietitians in the fall of 2008 to 2016. Questionnaires also included information on diet, lifestyle, environmental concerns, health and food security. TF items listed on the FFQ were drafted after consultations with community representatives to confirm the species available in each region. Open- and closed-ended questions gathered information about TF harvesting and production activities (including fishing, hunting, collecting plants, berries and seafood, and growing a garden) and TF consumption using a region-specific FFQ to estimate yearly/seasonal use of between 150 and 200 different types of TF. The FFQ required the participants to recall intake frequency seasonally over the past 12 months and data were presented by the reported frequency and number of days the food was reported in the past year. TF were categorized for reporting purposes into fish, seafood, land animals, birds, collected plants (including berries and maple syrup), and cultivated foods (e.g., beans, corn and squash).

Twenty-four-hour recalls used a 3-stage multiple pass method and portion sizes were estimated using 3-dimensional food models (Santé Quebec, Montreal, QC, Canada) (Batal et al. 2021). To assess TF quantities in grams consumed, reported frequencies from the FFQ were multiplied by the average daily serving size for each group of TF species (land animals, birds, fish, seafood, plants and cultivated plants) for each gender, age group and region as reported on the 24-h recalls.

An ecosystem framework (from West to East: Pacific Maritime, Boreal Cordillera, Montane Cordillera, Taiga Plains, Boreal Plains, Prairies, Taiga Shield, Boreal Shield, Hudson Plains, Mixedwood Plains and Atlantic Maritime (Fig. 1)) was used to capture TF patterns and make comparisons between ecozones (Chan et al. 2021; Wiken 1986).

As climate change has been recognized as having an impact on TF use (Ford 2012), participants in this study were asked open-ended questions to describe impacts on TF availability (i.e., less availability meant that the amount of TF available in the region to hunt, gather, etc. had decreased and fewer berry yields were due to hot, dry summers) that they specifically attributed to climate change. Other questions addressed the adequacy of TF equipment for gathering, barriers to TF use and benefits of TF. The responses to these open-ended questions were reviewed by a study analyst and categorized.

A multivariable regression was performed to assess the contribution of location (region and ecozone), road access and participant characteristics (age group, income source, employment, education level, self-reported health, body mass index status, and participation in TF harvesting activities) to variation in the number of days TF was eaten in the past year. The distribution of the number of days TF was eaten was right-skewed, so the square root of number of days of TF consumption, which was approximately normally distributed, was used as the dependent variable.

Data were entered into a study database using Epi Info 3.5.4 (Centers for Disease Control and Prevention, Atlanta, GA, USA, 1988). Data analysis used SAS/STAT version 9.4 (SAS, Cary, NC, USA, 2013). All analyses were weighted by community, household and individual for non-response and to ensure that they represented this population. Sample weights were adjusted for changes in population from 2008 to 2017, to reflect the population in 2017 (Chan et al. 2021).

## Results

**Fig. 2:**
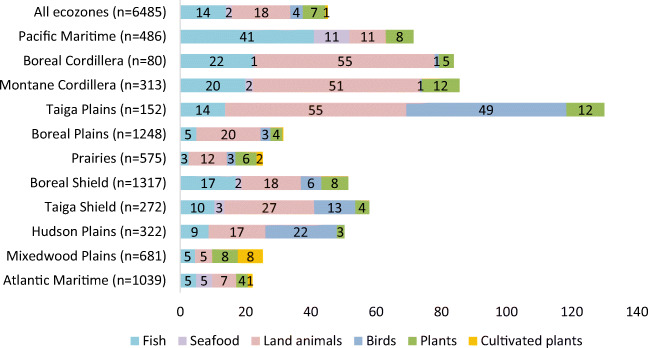
Grams of traditional food intake per day calculated from the food frequency questionnaire among First Nations adults living on reserves in Canada. Based on the reported daily frequency on the food frequency questionnaire multiplied by the average daily serving size for each group of TF species (land mammals, birds, berries, etc.) and each gender as reported in each region from the 24-h recalls

TF were preferred by most First Nations adults and they desired to consume them in larger quantities and more often. Across the regions, among all TF groups, land animals were consumed in the largest average amount (18 g/day), followed by fish (14 g/day), plants (wild and cultivated) (7 g/day), birds (4 g/day), and seafood (2 g/day) (Fig. 2). Land animals represented the largest proportion of TF food category in terms of grams consumed in most ecozones except in the Pacific Maritime and the Mixedwood Plains zones. In the Pacific Maritime ecozone, fish (58%) and seafood (15%) together contributed a greater share of the TF quantity compared with land animals (15%). In the Mixedwood Plains ecozone, plants (wild and cultivated) (62%) were the largest contributors. Land animals were reported to be consumed the most often per year (25 days), followed by fish (19 days), birds (9 days), plants (wild and cultivated) (8 days) and seafood (2 days). Table 1 provides the top 10 TF by ecozone. Moose was the most frequently available and consumed TF in Canada: it was the most or second most often reported in almost all ecozones except the Taiga Shield and Pacific Maritime ecozones, where it still appeared in the top ten.

**Table 1:** Top 10 most frequently consumed traditional foods by ecozone in First Nations adults living on reserves in Canada

Ecozone/rank	#1 (Number of days per year)	#2 (Number of days per year)	#3 (Number of days per year)	#4 (Number of days per year)	#5 (Number of days per year)	#6 (Number of days per year)	#7 (Number of days per year)	#8 (Number of days per year)	#9 (Number of days per year)	#10 (Number of days per year)
Pacific Maritime (*n* = 486)	Salmon	Eulachon /grease	Halibut	Seaweed	Fish eggs	Blackberry	Moose meat	Prawn	Crab	Deer meat
	62	23	16	15	14	13	12	9	8	8
Boreal Cordillera (*n* = 80)	Moose meat	Salmon	Trout	Balsam tree inner bark	Moose kidney	Caribou meat	Blueberries	Soapberry	Black bear fat	Moose liver
	109	56	10	8	8	8	7	7	6	6
Montane Cordillera (*n* = 313)	Moose meat	Deer meat	Salmon	Huckleberry	Soapberry	Labrador tea leaves	Elk meat	Saskatoon berry	Trout	Deer liver
	46	41	24	20	12	11	11	6	6	5
Taiga Plains (*n* = 152)	Moose meat	Ducks	Grouse	Northern pike	Mint leaves	Rat root	Geese	Rabbit	Saskatoon berry	Chokecherry
	96	78	19	16	15	15	13	11	10	9
Boreal Plains (*n* = 1248)	Moose meat	Mint leaves	Deer meat	Blueberries	Rat root	Walleye	Ducks	Elk meat	Saskatoon berry	Northern pike
	28	6	5	4	4	4	4	3	3	3
Prairies (*n* = 577)	Saskatoon berry	Moose meat	Deer meat	Elk meat	Chokecherry	Blueberry	Raspberry	Rat root	Mint leaves	Strawberry
	7	7	7	5	5	4	4	3	3	3
Taiga Shield (*n* = 272)	Labrador tea leaves	Caribou meat	Geese	Trout	Ptarmigan	Blueberry	Whitefish	Black bear fat	Grouse	Moose meat
	54	46	22	14	13	9	8	7	6	2
Boreal Shield (*n* = 1317)	Moose meat	Walleye	Blueberry	Geese	Whitefish	Raspberry	Ducks	Caribou meat	Northern pike	Strawberry
	20	15	10	6	6	4	4	3	3	3
Hudson Plains (*n* = 322)	Geese	Moose meat	Walleye	Caribou meat	Labrador tea	Northern pike	Ducks	Blueberries	Rabbit	Whitefish
	40	21	5	4	4	4	4	3	3	3
Mixedwood Plains (*n* = 681)	Corn	Beans	Deer meat	Squash	Maple syrup	Strawberry	Raspberry	Blueberry	Bird eggs	Walleye
	13	9	7	7	6	4	4	4	3	3
Atlantic Maritime (*n* = 1039)	Moose meat	Blueberry	Strawberry	Salmon	Raspberry	Fiddleheads	Haddock	Beans	Maple syrup	Trout
	12	7	5	3	3	3	3	3	3	2
Across ecozones	Moose meat	Salmon	Deer meat	Blueberry	Walleye	Labrador tea leaves	Geese	Raspberry	Strawberry	Ducks
	19	9	7	7	6	4	3	3	3	3

**Fig. 3:**
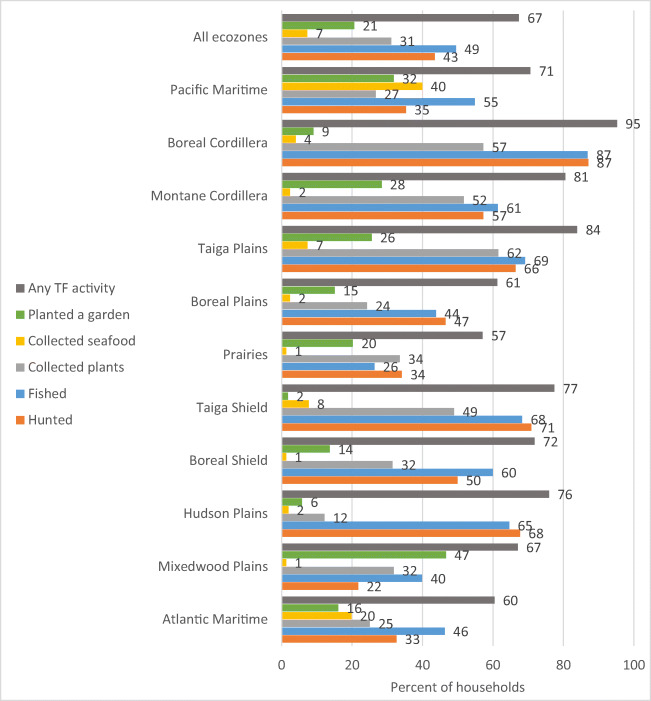
Types of traditional food harvesting and production practices reported at the household level by First Nations adults living on reserves in Canada by ecozone. Collected plants incorporates gathering of wild plants, including root digging and collection of maple sap

Diverse patterns of TF use were seen across the ecozones, with higher levels in Western and Northern regions and lower levels in Eastern and Southern regions. In multivariable analyses, TF intake was associated with location (highest in British Columbia and the Taiga Plains ecozone), engagement in harvesting activities, age group (higher in older individuals, i.e., 51 years of age and older) and gender (lower in women). Households reporting participation in TF harvesting activities had more TF use. Across Canada, 67% of households (range by region from 57% to 95% of households) were actively engaged in harvesting (Fig. 3).

Seventy-seven percent of participants identified that there were barriers to TF intake, with most indicating that they would have preferred more TF in their household. Of those who said there were barriers (*n* = 4354), in an open-ended question in which they were asked to list all the barriers to eating more TF, most (72%) identified one barrier, 22% identified two barriers and 6% identified three or more barriers. The three barriers mentioned most frequently across most ecozones were lack of a hunter in the household, lack of resources (i.e., money and/or equipment/transportation) and lack of time. Among the top barriers identified in 3 ecozones were availability (reported by 15.8% in the Pacific Maritime) and a lack of knowledge of harvesting (reported by 11.2% in the Mixedwood Plains and 10.6% in the Atlantic Maritime).

**Fig. 4:**
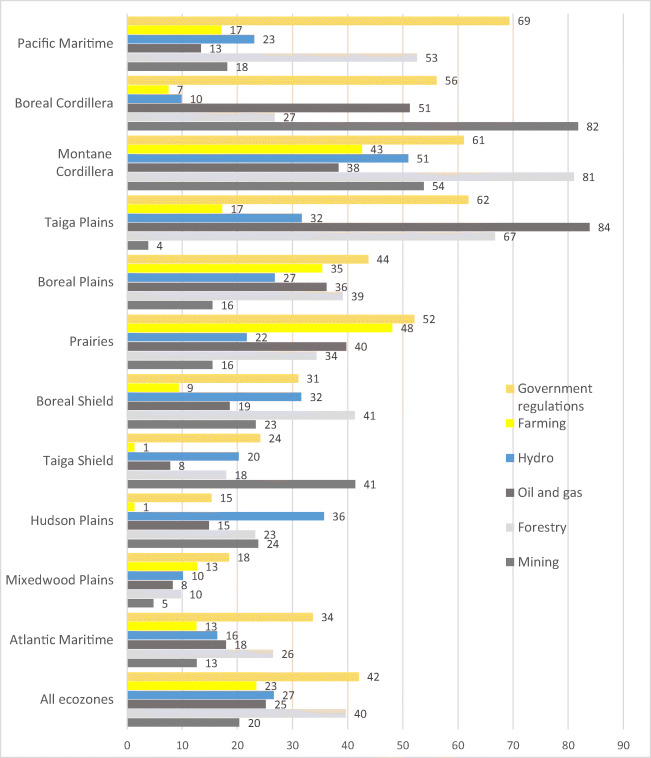
Factors impacting traditional food harvests as reported by First Nations adults living on reserves in Canada

Overall, 55% of participants said that the natural resource industries (mining, forestry, oil and gas, hydro, farming) affected their harvesting practices while 42% identified government regulations as a barrier. In the Boreal Cordillera, Montane Cordillera and Taiga Plains ecozones, more than 80% of adults indicated that natural resource activities negatively impacted their engagement in harvesting TF (Fig. 4).

In all ecozones, most adults said that they had noticed negative effects on TF that they attributed to climate change. Climate change was considered to impact both the overall amount of TF available and the ability to access TF. Changes to overall availability were mentioned more frequently by adults residing in the Pacific Maritime, Boreal Cordillera, Montane Cordillera and the Mixedwood Plains, whereas access challenges seemed to be more pronounced in the Hudson Plains and Taiga Shield. Participants reported that the top five benefits to TF were that it was healthy and nutritious (32%), natural and safe (19%), less expensive than MF (12%), tasted better (9%) and had cultural and educational importance/value (7%).

## Discussion

Traditional food is important to First Nations as it contributes to their spiritual, cultural and physical health (Willows 2005). TF is of superior nutritional quality compared with MF (Kuhnlein 2015; Kuhnlein and Receveur 1996; Batal et al. 2018). In order to promote the important practices of gathering and harvesting TF, it is important that we understand current practices and barriers to this activity. It is also critical to note that TF intake has been decreasing over time in Indigenous populations in Canada (Sheikh et al. 2011; Kuhnlein and Receveur 1996; Kuhnlein 2018; Kuhnlein et al. 2004) and that the current MF-based diet is poor and NRCD are highly prevalent (Batal and Decelles 2019; Batal et al. 2018).

We concentrated on the results from the FFQ because they gave us a better estimate of TF over time, so we could capture less commonly eaten foods only available during some seasons (Lavigne-Robichaud et al. 2018; Johnson-Down and Egeland 2013; Batal et al. 2005). Interviews for our study were conducted in the fall and this may have influenced the reported differences in land animals, fish, plants and birds as we were asking the participants to recall a full year of intake.

As we and others have established, TF practices are very different across regions in Canada (Kuhnlein et al. 2008). Because First Nations living on reserves were not included in national studies such as the CCHS, this is the first cross-Canada study for these Peoples below the 60^th^ parallel (Chan et al. 2021). We demonstrated a decrease in TF intake from North to South similar to other studies comparing Inuit with First Nations in the Yukon and the Northwest territories (Kuhnlein et al. 2001; Kuhnlein et al. 2008). In Cree communities in Northern Quebec, close to 100% of participants report TF consumption as did many communities in FNFNES (Johnson-Down and Egeland 2013). The First Nations Regional Health Survey reported between 70 and 76% of individuals often consuming TF (First Nations Information Governance Centre 2018). As would be expected, First Nations in marine zones were eating fish and seafood from the sea whereas those from more landlocked areas consumed more land animals.

As the promotion of TF is important in First Nations communities, we need to understand the factors that influence its use. We identified structural-level barriers to harvesting that are often out of the control of First Nations communities, such as industrial activities and government regulations that impact availability and harvesting practices. Household level barriers included insufficient resources to purchase/operate equipment, lack of a hunter and lack of time. Other studies of Indigenous Peoples in Canada have identified similar barriers to TF use (Chan et al. 2006; Laberge Gaudin et al. 2015; Skinner et al. 2006). TF systems are vulnerable to environmental changes, and climate change contributes to increasing vulnerability (Ford 2012).

Results from this study were statistically representative of the First Nations communities below the 60^th^ parallel across Canada. Random sampling and weighting of results contributed to the generalizability of this study to First Nations in the regions we targeted. It is possible that amounts of TF were overestimated by summing the intakes of different questions from the FFQ and because these were self-reports and TF is perceived as important culturally and health-wise (Willett 2012). It must be noted however that 60% of First Nations individuals do not live on reserves in Canada (Statistics Canada 2016), and although we can hypothesize that their TF use might be lower than reported here, we cannot speculate as to their intake of these important foods.

We recommend that new mechanisms be co-developed with First Nations to address weaknesses in current policy and program approaches to restore TF systems and support communities to increase their access to and use of TF. The importance First Nations place on TF from a holistic health perspective (spiritual, cultural, mental and physical) (King 2009) needs to be taken into account when devising programming for improved nutrition and health in First Nations communities, and dimensions of Indigenous food sovereignty need to feature in said programming. Empowerment of communities and tailoring of policies and regulations to allow optimal access and utilization of TF are needed. Our research has shown that many obstacles stand between First Nations and their preferred traditional foods. Adequate response to the public health crisis will have to tackle these obstacles in order to provide viable, sustainable, culturally relevant solutions. In an era of climate change and depleting natural resources, additional challenges of resource protection will also have to be central in any programming.
